# Supplementary material for: BIOMERO: A scalable and extensible image analysis framework
Source: Patterns (N Y). 2024 Jul 18;5(8):101024. doi: 10.1016/j.patter.2024.101024 (PMC11368678; doi:10.1016/j.patter.2024.101024)
Supplement: Document S1. Figures S1‒S8, Table S1, and Note S1 [file mmc1.pdf]

Patterns, Volume *5*

Supplemental information

BIOMERO: A scalable and extensible image analysis framework

Torec T. Luik, Rodrigo Rosas-Bertolini, Eric A.J. Reits, Ron A. Hoebe, and Przemek M. Krawczyk

Figure S1. Slurm folder structure, related to BIOMERO Python library

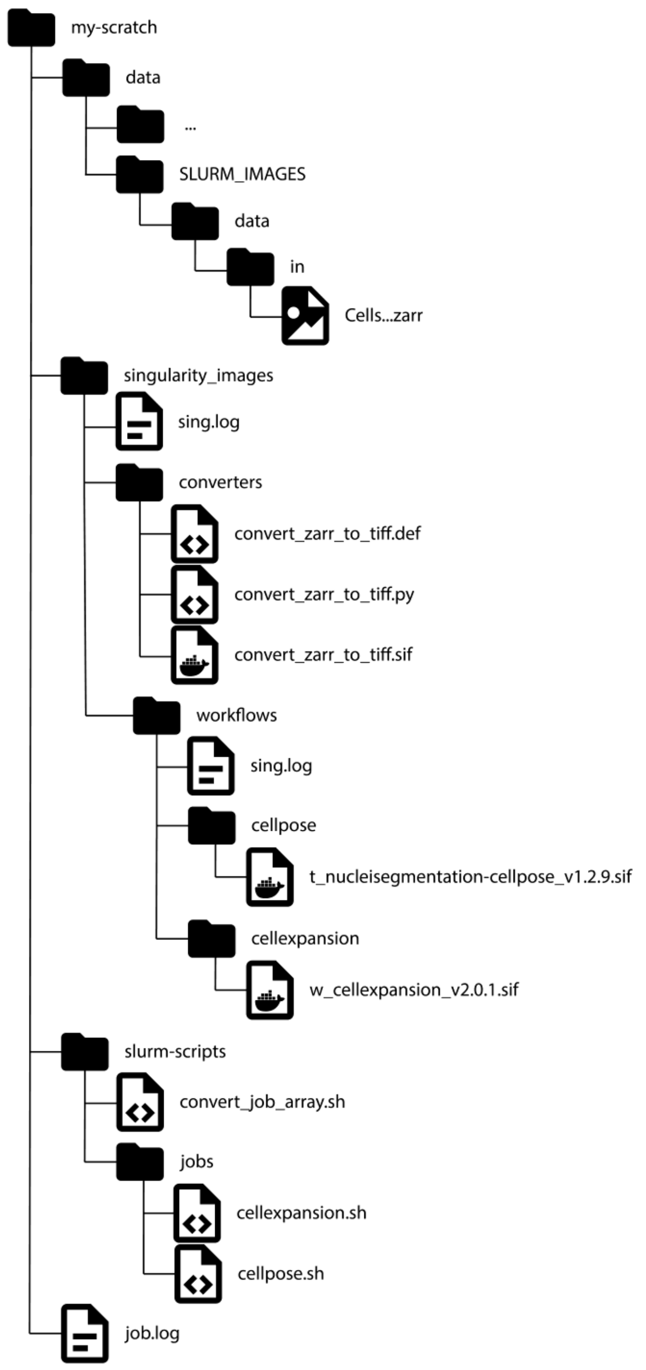

Figure S1 – Folder structure on Slurm nodes managed and created by BIOMERO. By design, all Slurm nodes share a storage in a folder ('my-scratch'), where logs of the running jobs are written. BIOMERO populates this storage with 3 folders: "singularity_images", "slurm-scripts" and "data". First

and foremost, “singularity_images” contains all container images for all workflows and converters, downloaded in the Singularity Image Format (SIF). “slurm-scripts” contains a Slurm job script for each workflow in the jobs subfolder. This folder could be pulled from Git instead of generated. Finally, “data” will contain the actual datasets transferred from OMERO (as zip files). Data will be unpacked into a folder structure required for BIAFLOWS workflows, with subfolders in(put), out(put) and g(round)t(ruth); only ‘in’ is shown. All these elements are automatically set up and can be refreshed by BIOMERO at any time when creating a connection.

Note S1. OME-NGFF, related to BIOMERO scripts and web interface.

For workflows to be FAIR, it is important to have 1 type of data (image) input. The Cytomine-0.1 apps from BIAFLOWS all expect TIFF files as input (and output). However OME is working on the OME-NGFF[1] (ZARR) to be a future data standard for bioimaging, especially designed for image data analysis. To support both directions, we are exporting all selected images from OMERO to the ZARR format, using the OMERO-CLI-ZARR (https://github.com/ome/omero-cli-zarr) app. On the Slurm cluster we run a converter from ZARR to TIFF format, using Slurm Array (https://slurm.schedmd.com/job_array.html) capabilities to parallelize and queue the extra computation on the high-performance hardware. This way we support current Cytomine-0.1 apps with TIFF but also open the door to future apps using ZARR. This is an extendable design where future workflows could use other converters, or (preferably) work directly on the ZARR data.

Figure S2. Dataset in OMERO, related to Example Use Case

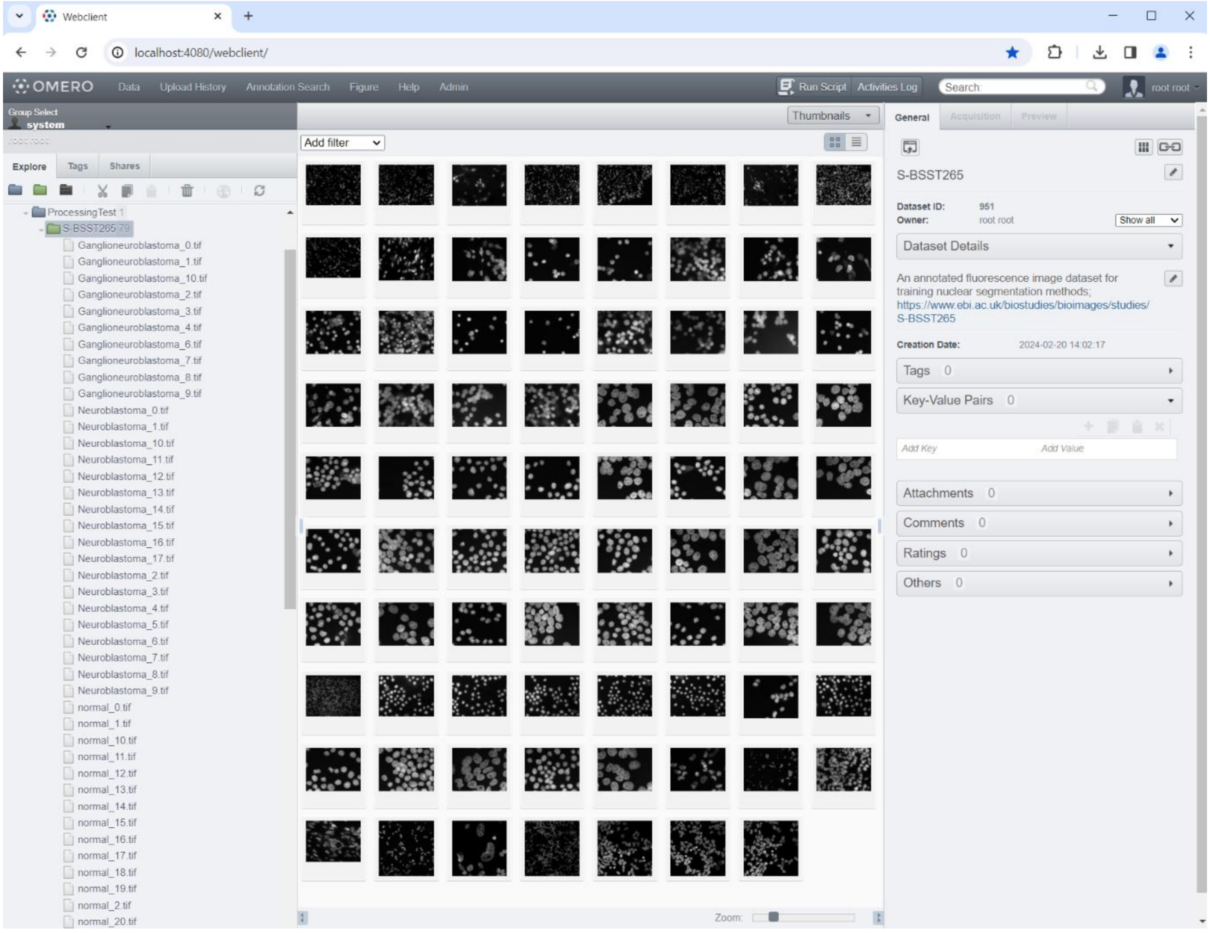

Figure *S2* – Screenshot of the OMERO user interface, with (the 79 raw images of) dataset S-BSST265 imported into a dataset. Ready to start analyzing with BIOMERO.

Figure S3. Select BIOMERO script in OMERO, related to Example Use Case

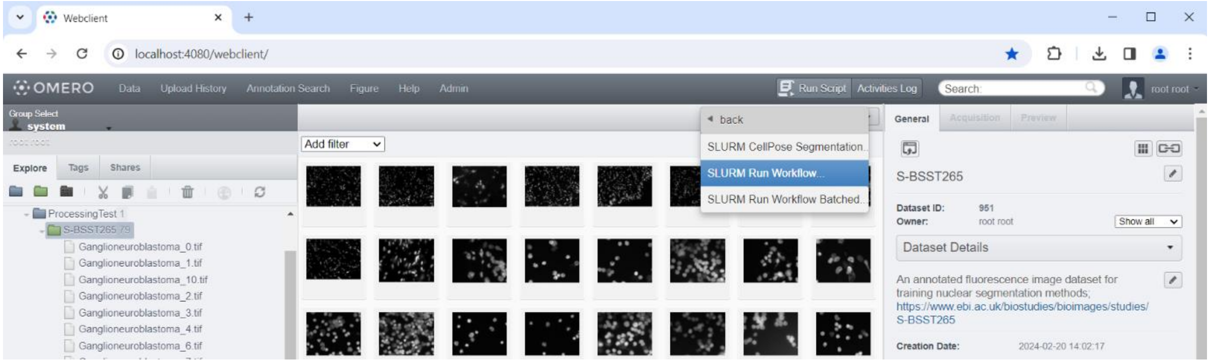

Figure S3 – Screenshot of the OMERO user interface, after clicking the Run Script button (note, this button is a cogwheel icon in default OMERO) and navigating to BIOMERO > workflows > SLURM Run Workflow. Note that you can preselect the dataset (shown in the left panel) to have it automatically selected as input data for the (BI)OMERO script.

Figure S4. BIOMERO script UI in OMERO, related to Example Use Case

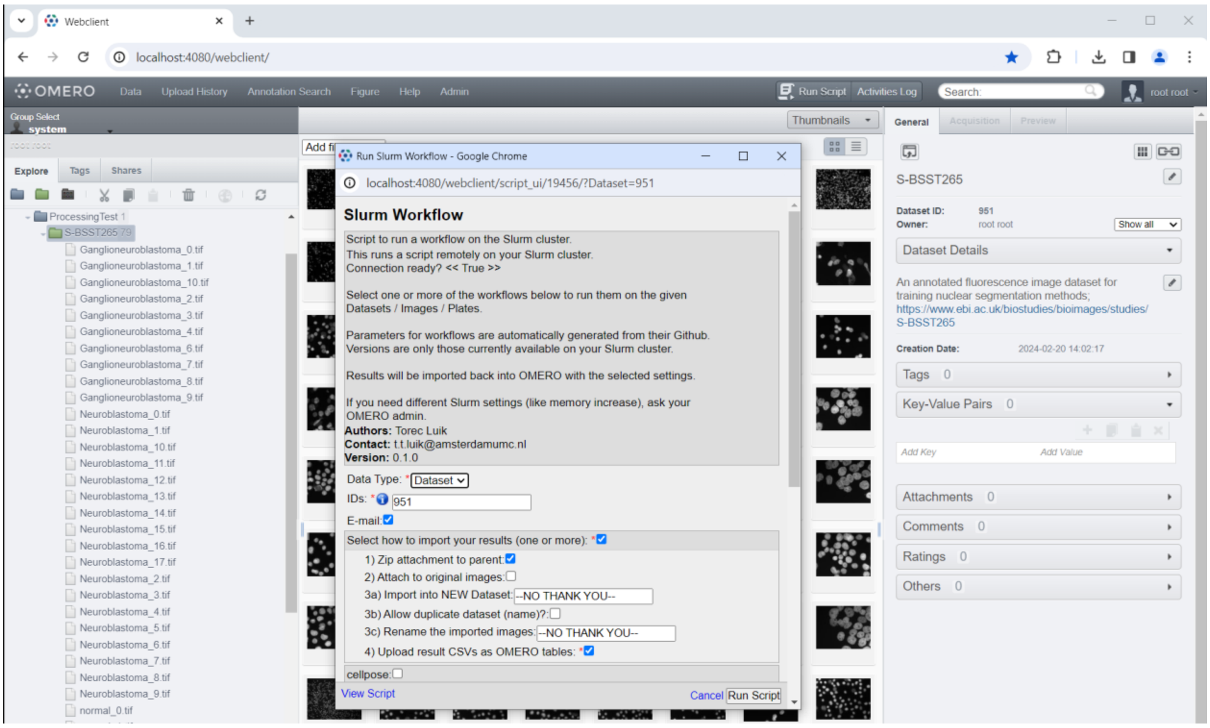

Figure *S4* – Screenshot of the OMERO user interface, after clicking the SLURM Run Workflow script option. The preselected dataset is added as input ID and the pop-up user interface shows options for how the user wants to import results back into OMERO. Further down the UI are the options for the configured BIOMERO workflows, here starting with ‘cellpose’.

Figure S5. Larger BIOMERO script UI in OMERO, related to Example Use Case

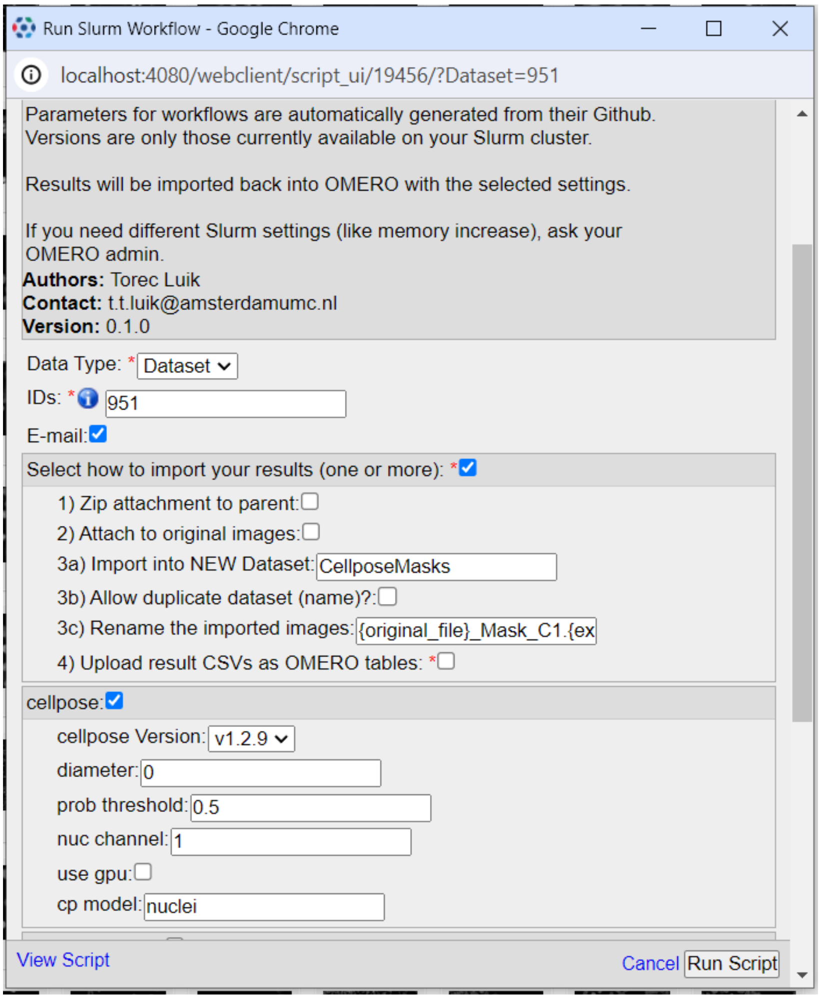

Figure S5 – Screenshot of the OMERO user interface, after clicking the SLURM Run Workflow script option, now zoomed in more. The preselected dataset is added as input ID and the pop-up user interface shows options for how the user wants to import results back into OMERO. We have selected to import the result (mask images from CellPose) into an OMERO dataset named 'CellposeMasks' and to rename these results to reference the image they originated from. Further down the UI are the options for the configured BIOMERO workflows, here we selected 'cellpose' and it showing all the parameters that are predefined in Cellpose's descriptor.json on its GitHub repository (generated on-the-fly). The version shown (v1.2.9) is a selection of container versions available on the connected Slurm cluster (queried when generating this user interface). We have selected to run CellPose on

channel 1 with the default ‘nuclei’ model, default threshold and default diameter (CellPose will guess the nucleus diameter per image), but not using GPU.

Figure S6. Processing BIOMERO script in OMERO, related to Example Use Case

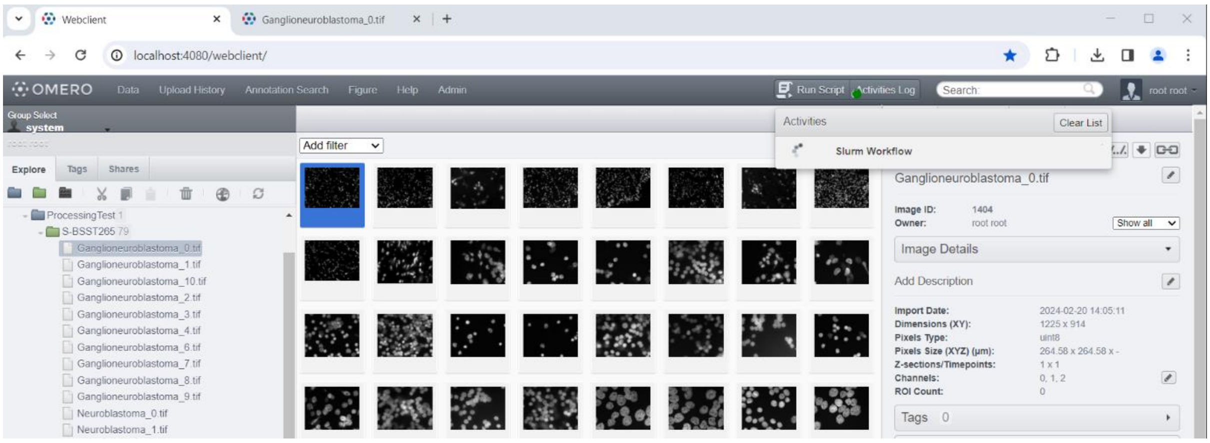

Figure S6 – Screenshot of the OMERO user interface, after running the SLURM Run Workflow script from Figure S5. In the background the script will perform all required operations, but the user will only see the rotating icon in the activity window until the workflow is done (completed, or erroneous).

Figure S7. Results of BIOMERO script in OMERO, related to Example Use Case

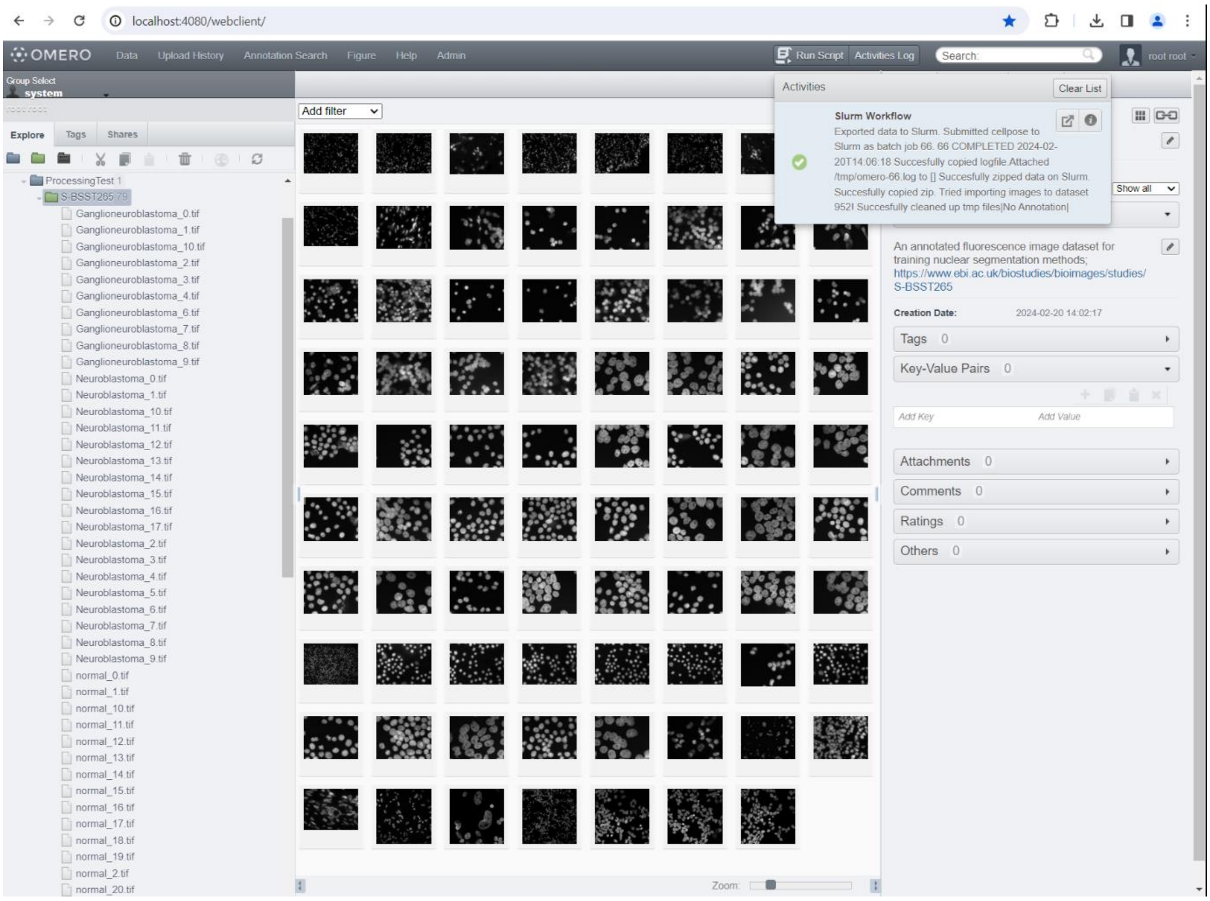

Figure S7 – Screenshot of the OMERO user interface, after running the SLURM Run Workflow script from Figure S5 and processing in Figure S6. A summary log is shown in the activity window, with a more detailed log attached from the BIOMERO script at the ‘I’ button. This is standard OMERO script behavior.

Figure S8. Resulting dataset of BIOMERO script in OMERO, related to Example Use Case

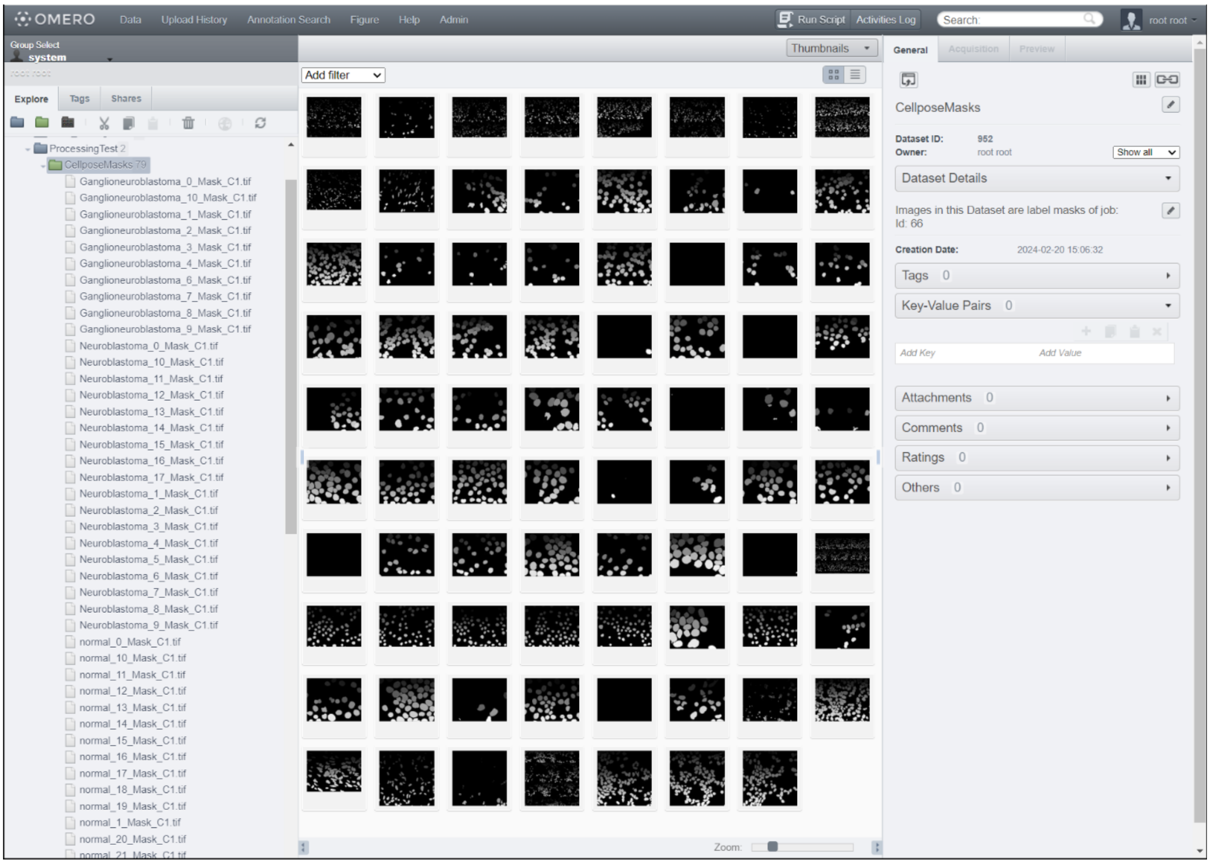

Figure S8 – Screenshot of the OMERO user interface, after the SLURM Run Workflow script from Figure S5 is completed successfully. A new dataset has been added (shown in the left panel), with all the mask images created by the CellPose workflow uploaded and renamed to match their origin image.

Table S1. Computation duration per job, related to Figure 4

Compute Configuration	Job id	Subjob	start	end	duration	Priority duration	Duration (min)	Priority duration (min)
Local 1 CPU	4308		14:00:58	20:06:28	06:05:30		365.5	
	5257		11:35:03	17:34:32	05:59:29		359.48	
	6206		10:10:23	16:19:14	06:08:51		368.85	
	7155		10:17:20	16:27:23	06:10:03		370.05	
Remote 1 GPU	265755		15:37:18	18:11:58	02:34:40		154.67	
	272945		13:54:49	15:45:29	01:50:40		110.67	
	275113		09:39:25	11:24:16	01:44:51		104.85	
	280745		11:16:16	13:14:04	01:57:48		117.8	
Remote 4 GPU 4 batches	264077	1	14:50:51	15:26:10	00:35:19		35.32	
		2	14:55:41	15:21:23	00:25:42		25.7	
		3	14:59:51	15:36:21	00:36:30		36.5	
		4	15:06:56	15:55:00	00:48:04		48.07	
		Total	14:50:51	15:55:00	01:04:09	00:48:04	64.15	48.07
	266608	1	09:40:10	10:08:05	00:27:55		27.92	
		2	09:43:43	10:06:07	00:22:24		22.4	
		3	09:47:27	10:17:09	00:29:42		29.7	
		4	10:06:09	10:37:10	00:31:01		31.02	
		Total	09:40:10	10:37:10	00:57:00	00:31:01	57.0	31.02
	271249	1	11:56:48	12:25:25	00:28:37		28.62	
		2	12:13:18	12:37:02	00:23:44		23.73	
		3	12:32:20	13:01:35	00:29:15		29.25	
		4	12:49:48	13:21:17	00:31:29		31.48	
		Total	11:56:48	13:21:17	01:24:29	00:31:29	84.48	31.48
	273440	1	07:42:50	08:09:29	00:26:39		26.65	
		2	08:09:51	08:31:46	00:21:55		21.92	
		3	08:31:51	09:00:04	00:28:13		28.22	
		4	09:00:51	09:29:50	00:28:59		28.98	
		Total	07:42:50	09:29:50	01:47:00	00:28:59	107.0	28.98

Table S1 – Computation duration per job on different Slurm configurations. The start and end are queried from the Slurm accounting ('sacct') information and the durations and minutes are calculated from these times. The ‘Remote 4 GPU 4 batches’ jobs have subjobs that are indicated with ids 1,2,3,4 and are aggregated in 2 different ways in the ‘Total’ row: the duration between the start of the first job and end of the last job; and the duration of the longest of its subjobs (called the Priority duration). The Boxplot in Figure 4 shows the distribution of these job durations in minutes, with batches only using the Total row and splitting out duration and priority duration into 2 boxes.

Supplemental References

1. Moore, J., Allan, C., Besson, S., Burel, J.-M., Diel, E., Gault, D., Kozlowski, K., Lindner, D., Linkert, M., Manz, T., et al. (2021). OME-NGFF: a next-generation file format for expanding bioimaging data-access strategies. Nat Methods *18*, 1496–1498. 10.1038/s41592-021-01326-w.
