# Supplementary material for: BIOMERO: A scalable and extensible image analysis framework
Source: Patterns (N Y). 2024 Jul 18;5(8):101024. doi: 10.1016/j.patter.2024.101024 (PMC11368678; doi:10.1016/j.patter.2024.101024)
Supplement: Document S2. Article plus supplemental information [file mmc2.pdf]

Descriptor

Patterns

BIOMERO: A scalable and extensible image analysis framework

Highlights

- Helping microscopy users focus on research with easy access to image analysis
- Connecting image data management and advanced analysis for microscopy
- Enhancing collaboration and data sharing for microscopy researchers
- Removing barriers to image analysis to advance microscopy research

Authors

Torec T. Luik, Rodrigo Rosas-Bertolini, Eric A.J. Reits, Ron A. Hoebe, Przemek M. Krawczyk

Correspondence

t.t.luik@amsterdamumc.nl (T.T.L.), p.krawczyk@amsterdamumc.nl (P.M.K.)

In brief

BIOMERO introduces a user-friendly framework to simplify image analysis for microscopy users, addressing the complexities of managing and analyzing large image datasets. By integrating with high-performance computing, BIOMERO empowers researchers to focus on biological research while streamlining workflows and facilitating collaboration. This transformative tool bridges the gap between image data management and analysis, advancing scientific discovery in microscopy research and beyond.

Check for updates

Luik et al., 2024, Patterns *5*, 101024
August 9, 2024 © 2024 The Authors. Published by Elsevier Inc.
https://doi.org/10.1016/j.patter.2024.101024

CellPress

Patterns

CellPress
OPEN ACCESS

Descriptor

BIOMERO: A scalable and extensible image analysis framework

Torec T. Luik,[1,*] Rodrigo Rosas-Bertolini,[1] Eric A.J. Reits,[1] Ron A. Hoebe,[1,2] and Przemek M. Krawczyk[1,2,3,*]
[1]Amsterdam UMC, Department of Medical Biology, Amsterdam, North-Holland 1105AZ, the Netherlands
[2]These authors contributed equally
[3]Lead contact
*Correspondence: t.t.luik@amsterdamumc.nl (T.T.L.), p.krawczyk@amsterdamumc.nl (P.M.K.)
https://doi.org/10.1016/j.patter.2024.101024

THE BIGGER PICTURE Microscopy is indispensable for exploring the intricacies of biology in scientific research. However, the analysis of microscopy image data often entails complex workflows and technical hurdles to execute them, especially with the emergence of deep learning. We introduce BIOMERO, a transformative extension of the popular OMERO image database, that simplifies image analysis for microscopy users.

SUMMARY

In the rapidly evolving field of bioimaging, the integration and orchestration of findable, accessible, interoperable, and reusable (FAIR) image analysis workflows remains a challenge. We introduce BIOMERO (bioimage analysis in OMERO), a bridge connecting OMERO, a renowned bioimaging data management platform; FAIR workflows; and high-performance computing (HPC) environments. BIOMERO facilitates seamless execution of FAIR workflows, particularly for large datasets from high-content or high-throughput screening. BIOMERO empowers researchers by eliminating the need for specialized knowledge, enabling scalable image processing directly from OMERO. BIOMERO notably supports the sharing and utilization of FAIR workflows between OMERO, Cytomine/BIAFLOWS, and other bioimaging communities. BIOMERO will promote the widespread adoption of FAIR workflows, emphasizing reusability, across the realm of bioimaging research. Its user-friendly interface will empower users, including those without technical expertise, to seamlessly apply these workflows to their datasets, democratizing the utilization of AI by the broader research community.

INTRODUCTION

In the realm of modern bioimaging, efficient management and analysis of large image datasets stand as pivotal challenges. The open-source bioimaging platform OMERO[1] excels in image data management, supporting all major proprietary microscopy data and metadata formats and allowing users to view and annotate their images in a web browser. OMERO is frequently used by core facilities in the life sciences to store and manage microscopy data for their users. While OMERO supports Python scripts, it currently lacks native capabilities for conducting (remote) data analysis. The prevailing approach involves exporting data from OMERO to user-installed applications, for example, Fiji[2] or QuPath,[3] resulting in the analysis computation occurring on the user's end rather than within the integrated data management suite. However, utilizing the simple Python script capabilities on the OMERO server also falls short of addressing crucial aspects: the lack of resource management, such as CPU, GPU, and memory utilization, due to the absence of a robust queue mechanism within OMERO, and the inherent clash of hardware requirements between data management, image viewing, and image analysis. This poses a question not only about the scalability of compute power for larger images or datasets but also about the reproducibility of the image analysis conducted using such applications, especially when considering high-throughput applications like high-content or high-throughput screening (HCS/HTS). The FAIR (findable, accessible, interoperable, and reusable) principles[4] have provided a guideline for data management, and recent advancements have also ported these guidelines to (research) software[5] and toward FAIR workflows.[6] By adding metadata (schemas), hosting them in public repositories, supporting standard file formats, and containerizing the execution environments, major steps toward such FAIR image analysis workflows can be achieved.

Hence, we identify two major challenges: first, the need for cohesive integration and orchestration of FAIR workflows from

This is an open access article under the CC BY license (http://creativecommons.org/licenses/by/4.0/).

OMERO, and second, the ability to harness high-performance computing (HPC) resources effectively for analyzing big image datasets, especially in HCS/HTS contexts.

The landscape of existing solutions consists of several tools addressing specific aspects of bioimaging challenges.

(1) First and foremost, the Slurm Workload Manager, often simply referred to as Slurm, is a job scheduler available on many of the world's high-performance clusters (65% of the TOP500; https://www.schedmd.com/slurm/), which enables efficient distribution of workflow workloads over multiple computers. To use it, however, one needs to log in to a specific server using a command-line interface, download all the required data and tools, and then run jobs scripts with a specific hardware configuration, which requires specialist computer science knowledge not prevalent among researchers.
(2) Cytomine[7] specializes in managing whole-slide imaging data and digital pathology but lacks support for HCS/HTS and is less used in general microscopy core facilities. Cytomine does, however, excel in the scalable execution of modular Cytomine apps (workflows), integrated into the Cytomine user interface (UI), by executing them on an HPC cluster with Slurm while abstracting these operations away from the user.
(3) BIAFLOWS[8] is an extension of Cytomine, funded and developed by NEUBIAS to support reproducible workflows across bioimaging. It particularly enables comparison of workflow results and increases interoperability of such workflows by also enabling execution outside of the Cytomine environment. Neither Cytomine nor BIAFLOWS, however, offers integration with OMERO, making it very difficult to execute their workflows on OMERO-stored data. However, the (methods of packaging) workflows are a considerable step toward FAIR workflows.
(4) "ObiWan-Microbi"[9] is a recent tool focusing on microbe colony and segmentation with the ability to execute workflows from its own model zoo and offload computations to another server. While it integrates segmentation features with OMERO, it falls short in accommodating a diverse range of FAIR containers, such as BIAFLOWS apps. Notably, it lacks support for container technology and efficient offloading to HPC clusters, particularly missing the queueing capabilities of Slurm.
(5) OMERO Plus (https://www.glencoesoftware.com/products/omeroplus/), a proprietary version by Glencoe Software, provides extra computational capabilities integrated with OMERO, including the OMERO-CellProfiler Connector (https://www.glencoesoftware.com/solutions/hcs/) for remote execution of CellProfiler pipelines via OMERO clients on different HPC systems and cloud platforms. However, it also lacks support for more generic FAIR workflows and, more importantly, is not free and not publicly available to the bioimaging community.
(6) The Fractal Analytics Platform (https://fractal-analytics-platform.github.io/), a tool still in its early stages and under active development, focuses on workflow computations on ZARR files only. However, it lacks direct integration with source data and UIs and requires managing a separate server/client architecture next to OMERO. Moreover, it also does not support generic FAIR containerized workflows, like BIAFLOWS apps.

To enable both execution of FAIR workflows and HPC support in an OMERO environment, we present BIOMERO, bioimage analysis in OMERO. BIOMERO is an open-source framework aimed at integrating bioimaging data storage and management with high-throughput, FAIR image analysis pipelines. BIOMERO consists of the BIOMERO[10] Python library and tools for its integration with the OMERO UI, the BIOMERO scripts. BIOMERO extends the capabilities of OMERO, providing efficient access to HPC resources and enabling scalable, FAIR workflows in the bioimaging field. By supporting BIAFLOWS workflows, BIOMERO fills a critical gap in existing infrastructure, facilitating the execution of intricate bioimaging analyses and unifying OMERO and the Cytomine/BIAFLOWS platforms under a common umbrella of FAIR workflows.

RESULTS

Architecture and design

BIOMERO is a framework that connects OMERO and HPC, redefining the architecture of bioimaging workflows for enhanced scalability and promoting the adherence of such workflows to FAIR principles. BIOMERO is a combination of the BIOMERO Python library and a set of BIOMERO scripts that allow user interaction from within the OMERO web interface. To make use of BIOMERO, an existing OMERO server and an existing Slurm HPC cluster need to be available.

Figure 1 shows an overview of the BIOMERO architecture. At the core, there is the BIOMERO Python package installed by the administrator on the OMERO server, providing an application programming interface (API) to Slurm on the HPC cluster. This interface can be accessed from BIOMERO scripts, which regular users can run from OMERO web interface (OMERO.web). From the web interface, the user can then run a BIOMERO script (A) that interfaces with the BIOMERO library (B) to send OMERO data to HPC and start analysis jobs on the HPC cluster (C), poll their progress (D), and retrieve results to store back in OMERO (E). With BIOMERO, users do not need to be versed in Slurm commands, data transfer procedures, or the specifics of Docker container and application codes. BIOMERO abstracts most of the data and workflow management, ensuring that a wider range of users can leverage the distributed compute power directly from the OMERO web interface to run predefined workflows (e.g., segmentation) on their data.

BIOMERO Python library

The BIOMERO Python library has been released on GitHub with the permissive Apache 2.0 license and published on PyPI for easy installation on OMERO servers. The package contains a main class, the *SlurmClient*, which serves as the API to a connected HPC cluster running Slurm. It builds upon the Python library Fabric (https://www.fabfile.org/), which enables running commands on remote machines through a secure shell (SSH, https://www.ssh.com/academy/ssh) connection; this allows BIOMERO to connect the OMERO server to any remote Slurm

CellPress
OPEN ACCESS

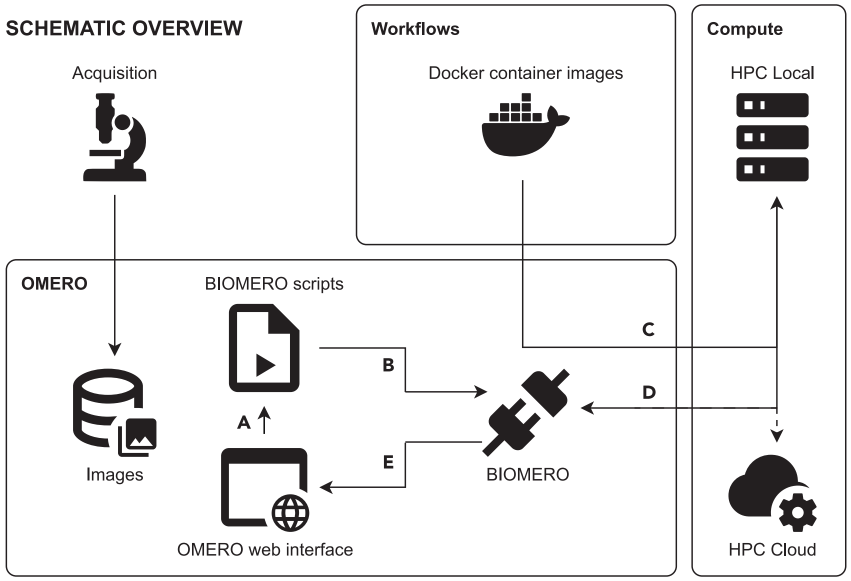

Figure 1. BIOMERO manages connection between OMERO and HPC (local or cloud)
At the center, we have the open-source BIOMERO Python library. Users tell BIOMERO what workflow to run on what images by selecting one of BIOMERO scripts (A), available in the OMERO web interface. The OMERO script will communicate with the BIOMERO library (B) to move data and track workflow execution. BIOMERO downloads (if needed) and executes the required Docker containers (C) as jobs on the HPC (local or cloud). The job status and logs from the HPC executions are polled through the BIOMERO library (D), and once the job is completed, results are stored back in OMERO (E) and communicated to the user via the OMERO web interface.

cluster that can be accessed with SSH, whether in the cloud, locally, or at a centralized data center. BIOMERO also adds an abstraction layer via dedicated Python functions, covering all major parts of the workflow life cycle.

The library is configured by the "*slurm-config.ini*" file, where the OMERO administrator can describe what workflows should be available to end users and where to find them online (GitHub repository and version), as well as the SSH settings for the target Slurm cluster. Once configured, the *SlurmClient* class can connect to the existing Slurm cluster and provides a function to automatically configure a managed folder structure (see Figure S1), including downloading the container images for the workflows.

Once the Slurm environment is ready, it can execute the preconfigured workflows as jobs. To run a workflow as a job, Slurm requires a job script specifying the required hardware, container image, and job-related parameters such as input folder and workflow settings. The Slurm job script will, by default, be generated by BIOMERO from the workflow's metadata (the descriptor file) stored on GitHub but can either be adjusted partially or entirely pulled from a GitHub repository (both configurable in the *slurm-config.ini* file). A full list of commands and functions available from the BIOMERO library can be found in the latest documentation (https://nl-bioimaging.github.io/biomero/).

BIOMERO scripts and web interface

The second part of BIOMERO entails a set of BIOMERO scripts. We enhance OMERO's existing scripting capabilities, providing an intuitive way for users to interact with and execute configured workflows directly from the OMERO web interface. These BIOMERO scripts use the BIOMERO library, described previously, to run workflows as Slurm jobs, poll the jobs, and, most importantly, get data back and forth from OMERO to the Slurm cluster. We have provided example implementations for running workflows in OMERO web interface (OMERO.web) through several example scripts published on GitHub (https://github.com/NL-BioImaging/biomero-scripts). This system is highly extensible and modular, and we encourage the community and OMERO administrators to create their own scripts for their specific workflow or UI needs.

BIOMERO scripts that we have provided are shown below.

- *SLURM Init Environment*," which will take care of creating the setup in Figure S1 based on the configuration file.
- *SLURM Run Workflow Batched*," which allows users to run any of the configured workflows on select input images, datasets, or plates. It creates a job for every batch of input images to run in parallel on Slurm, and it will poll execution of these jobs and only return when all jobs are finished. It uses the parameters read from the workflow descriptor to create UI elements for OMERO web.
- "*SLURM Run Workflow*," which is called by *Batched* per batch, to run any of the configured workflows as one Slurm job on all provided input images, datasets, or plates. It calls the scripts "*SLURM Image Transfer*" to transfer data from OMERO and, when the Slurm job has finished, "*SLURM Get Results*" to get the results back into OMERO.
- *SLURM Image Transfer*, an adaptation of OME's "*Batch Image Export*" script (https://github.com/ome/omero-scripts/blob/develop/omero/export_scripts/Batch_Image_Export.py), to export images from OMERO as ZARR and transfer them to the HPC using secure copy protocol (https://www.ssh.com/academy/ssh/scp). See Note S1 for a discussion on using ZARR here.
- *SLURM Get Results* can upload a folder from HPC back into OMERO as images in a dataset, attachments to selected input files, or a generic zip attachment to a parent project.

Executing FAIR workflows from OMERO

BIOMERO advocates for FAIR principles in bioimaging research through the execution of BIAFLOWS workflows. Leveraging publications on GitHub and DockerHub, metadata descriptors, and Docker or Singularity containerization, BIAFLOWS workflows facilitate the adoption of FAIR practices.[6] BIAFLOWS workflows are essentially derived from Cytomine apps but offer

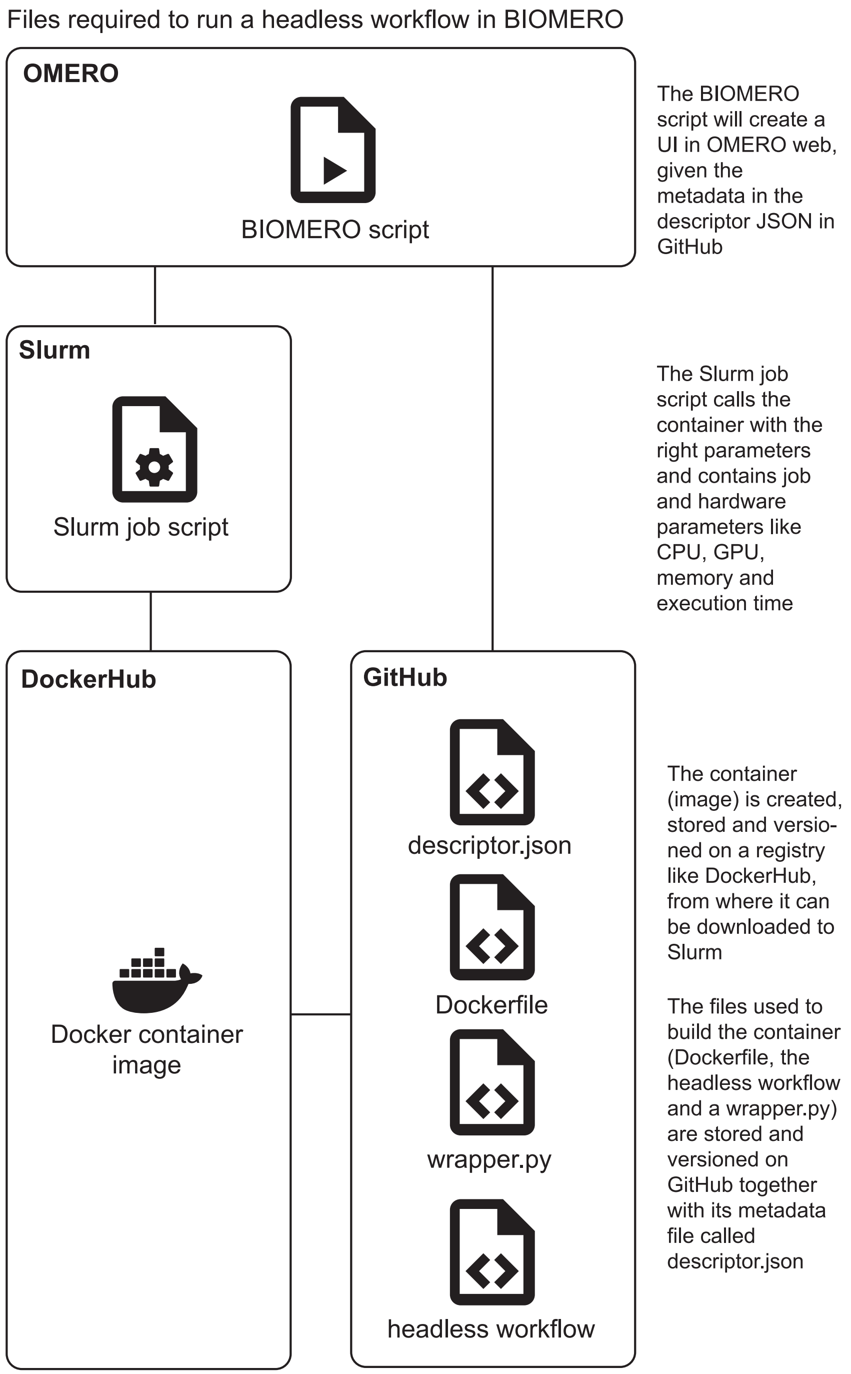

Figure 2. Files required to run a workflow in BIOMERO

The underlying executable code needs to be a headless program (i.e., it does not require a graphical UI). The input parameters should be described in a configuration file called descriptor.json, and any preprocessing (or file reading) can be added to the wrapper.py, while the required environment should be defined (Python version, Python libraries, Fiji plugins, MATLAB packages, OS, etc.) in the Dockerfile. These files should be published and versioned on GitHub (or another public online platform). From the Dockerfile, a container image should be built, versioned, and hosted on a public online container registry like DockerHub to ensure reproducibility. At this point, anybody can pull the workflow to their local computer and run the workflow using Docker, Podman, Singularity, or a dedicated workflow management system. Finally, to allow execution on HPC from OMERO, BIOMERO requires a Slurm job script that defines hardware parameters (we will generate a default one if none are provided) and a BIOMERO script that will generate the UI in OMERO.web (we provide a generic implementation of such an OMERO script that leverages the descriptor.json) and call on the BIOMERO library. If these are all in place, then the user can execute the workflow in OMERO via the script UI.

enhanced FAIR attributes, especially in their capacity to be executed independently of Cytomine, emphasizing increased interoperability. In conclusion, BIOMERO seamlessly supports BIAFLOWS workflows, and with minor adjustments, it can also accommodate Cytomine apps or any FAIR workflow with other metadata descriptors.

In Figure 2, we show the files required to make any workflow compatible with the BIOMERO framework. Any executable code that can run headless (i.e., without a graphical UI) from the command line (e.g., a Fiji Macro, a compiled MATLAB script, a Python script, an R script, a .exe file) should be packaged up by adding 3 files: a Dockerfile to define the environment in which the executable code can run (e.g., OS, Python version, Python libraries, Fiji plugins), a descriptor JSON file that defines the metadata of the code (e.g., command-line parameters, what values they take and their descriptions), and a short Python script that wraps the executable code (running it as a Python subprocess) and provides it with the correctly preprocessed input and output data. These 4 files should be published online on a public code versioning repository (e.g., on GitHub). With these files in place, a Docker container image can be built, which will allow the executable code to be executed from any platform with container software like Docker, Podman, or Singularity installed. However, to ensure reproducibility, the built container image should also be published and versioned in a public container registry like DockerHub. This process can be automated, via GitHub Actions, for instance, to keep versions of GitHub and DockerHub the same. At this point, the workflow is interoperable and reproducible in general, and it can be registered in BIOMERO's configuration file. To execute it from BIOMERO, we require two more files for which BIOMERO provides sensible defaults: the Slurm job script to run the container on Slurm with the right hardware parameters (e.g., memory, CPU, GPU, and execution time) and the

BIOMERO script to create a UI in OMERO and connect with the BIOMERO library. However, OMERO administrators are also encouraged to make their own custom scripts for either of these, as the Slurm job script defines the hardware requirements for the workflow (e.g., whether it needs a GPU or not) and the BIOMERO script defines a UI and handles input/output from OMERO.

Slurm job scripts

To schedule any workflows in Slurm, they require a Slurm job script. These scripts mainly describe the computational resources required (i.e., memory, CPU, and/or GPU) and the command to execute the workflow with the right parameters. Note that BIOMERO already provides default Slurm job scripts for every workflow configured. By default, no action will be required by the user or OMERO administrator to set up a Slurm job script, but we do allow providing custom scripts or customizing the default scripts from the BIOMERO configuration file.

In Figure 3, we show a detailed overview of all the layers of execution that are required (and automatically taken care of by our framework) to execute FAIR workflows from OMERO on HPC. The Slurm job script is what is actually submitted to the Slurm queue and assigned and executed on the desired compute nodes. Inside the Slurm job script, we use Singularity to run the Docker container on the input images. Another step handled by a Slurm job script is the conversion from ZARR to TIFF files, as all BIAFLOWS containers require TIFF input; yet, we extract ZARR from OMERO. For efficiency, the conversions are also added in parallel to the Slurm queue, allowing the use of HPC infrastructure for speedier computation. Obviously, such conversion can be omitted when using workflows that natively execute on ZARR files.

One of the important factors not handled by the FAIR workflow setup from BIAFLOWS is the Slurm job script and its hardware parameters. Other workflow descriptor schemas like "boutiques"[11] do allow defining hardware parameters as part of the workflow metadata. BIOMERO provides sensible defaults and allows further customization of these parameters from its configuration file. We decided not to add these parameters to the OMERO UI to hide HPC implementation details from the end user.

Example use case: CellPose from OMERO

Among the major use cases for bioimage analysis is the segmentation of cells or nuclei from microscopy images. One of the most popular nuclei segmentation algorithms is CellPose.[12] This algorithm is also packaged as a FAIR workflow by BIAFLOWS and thus available in BIOMERO. To showcase an application of BIOMERO in OMERO, we have imported the raw images from dataset S-BSST265,[13] a fluorescence image dataset collected for nuclear segmentation, from the BioImage Archive[14] into OMERO as a dataset and set up CellPose for BIOMERO. To create a more representative dataset size, we have duplicated it 12 times to get 948 images total. We show screenshots of the input, output, and steps taken in OMERO (as the user) to run CellPose with BIOMERO in Figures S2–S8. We also show how leveraging HPC can help reduce the processing time of such image analysis workflows in Figure 4, where we compare the computation times of a local run (on our OMERO workstation acting as a private Slurm CPU node as "local 1 CPU") with our remote HPC cluster. For the HPC cluster, we show 3 options, with up to 4 GPUs: First, "remote 1 GPU" executes the whole workflow on 1 GPU node (similar to "local 1 CPU"). Second, "remote 4 GPU 4 batches" splits the job into 4 batches and executes each on a separate GPU node. Finally, we also show the "priority" computation time where each batch would be assigned to a GPU node at the same time for maximum efficiency. Because we do not control the HPC cluster in our experiment, the batches normally often have to wait in the queue for other jobs to finish first and so do not run perfectly in parallel. While this reasonably shows the potential of BIOMERO in leveraging HPC compute, note also that this is both a simplified and a limited view: simplified because there are a lot of practical factors that could impact the actual lead time for the user (e.g., waiting in the queue of a busy HPC cluster, uploading results back into OMERO) and limited because, theoretically, you could scale HPC up to hundreds of GPUs (and small batches to divide among the nodes). See Table S1 for a full view of the job and sub-job run times.

Tutorials

We host a number of FAIR Cytomine-0.1 workflows on GitHub, which we have updated and tested with BIOMERO. We have also included a tutorial for the creation of several of these containers, noted in the list below.

- CellPose,[12] hosted at https://github.com/TorecLuik/W_NucleiSegmentation-Cellpose.
- CellProfiler for spot counting (https://github.com/tischi/cellprofiler-practical-NeuBIAS-Lisbon-2017/blob/master/practical-handout.md), hosted at https://github.com/TorecLuik/W_SpotCounting-CellProfiler.
  - Tutorial available at https://nl-bioimaging.github.io/biomero/tutorial_link.html#cellprofiler-tutorial.
- CellExpansion, hosted at https://github.com/TorecLuik/W_CellExpansion.
  - Tutorial available at https://nl-bioimaging.github.io/biomero/tutorial_link.html#cellexpansion-tutorial.
- Spot-counting script, hosted at https://github.com/TorecLuik/W_CountMaskOverlap.
- Part of the same tutorial as CellExpansion.

To make it easier to get started with BIOMERO, we have also hosted two tutorials for setting up a simple Slurm cluster to connect your OMERO to a local computer using either

- Docker, https://nl-bioimaging.github.io/biomero/tutorial_link.html#local-slurm-tutorial, or
- Google Cloud, https://nl-bioimaging.github.io/biomero/tutorial_link.html#google-cloud-slurm-tutorial.

DISCUSSION

In conclusion, our open-source framework paves the way for more widespread accessibility to FAIR bioimaging workflows, fostering collaboration and advancing the capabilities in bioimaging research. We promote best practices by funneling workflows in our BIOMERO into a FAIR shape and reusing workflows from Cytomine/BIAFLOWS, but we also enable a lot more users to execute these workflows by integrating with the OMERO web

interface. We present a Python library enabling integration of OMERO and Slurm, allowing improved scalability and efficiency in bioimaging workflow execution and promoting best computation practices.

We want to note that our BIOMERO is modular, extensible, and permissively open source, so while we have provided a first integration with OMERO using BIOMERO scripts, other implementations are also possible and encouraged. Cytomine itself is also still actively being developed at the time of writing and will likely improve upon the BIAFLOWS containers. We will incorporate such improvements in future versions of BIOMERO.

Current limitations are mostly related to our workflow design being based on BIAFLOWS workflows: support for any generic container workflow is limited to only interpreting metadata values in the metadata descriptor JSON file that corresponds to the Cytomine-0.1 schema (https://doc.uliege.cytomine.org/dev-guide/algorithms/descriptor-reference). We also assume at this point that each workflow will accept an input folder of 8-bit/16-bit TIFF (2D) or single-file OME-TIFF (C,Z,T) images. Both are current implementations based on BIAFLOWS workflows but are not inherent to our framework, and more use cases can and will be supported in the future. A third limitation is the OMERO.scripts interface that we use for BIOMERO scripts. The two main blocking features are the static interface, disallowing user interactivity, and the absence of progress feedback: the user is only notified when the (long-running) process is finished. A final limitation is that transferring large datasets (e.g., terabytes) to a remote Slurm cluster might be a costly operation by itself, depending on the connectivity between the servers.

The next steps

In future versions, we will support more (generic) workflow descriptor languages besides Cytomine-0.1. We suggest supporting boutiques (on which Cytomine-0.1 is based) and/or the common workflow language.[15] One of the major features that would be useful from such extended schemas is the description of required computational resources, which should be integrated into the generated Slurm job script.

We also plan to implement and support workflow management systems or languages to chain modules together in a structured fashion, for example, SnakeMake,[16] NextFlow,[17] MLFlow (https://mlflow.org/), or even Fractal (https://fractal-analytics-platform.github.io/).

Moreover, we aim at supporting OME-NGFF[18] workflows instead of being limited to TIFF input. This should be relatively straightforward since we already export images as ZARR. However, we are also aware that OME-NGFF is designed to be remotely accessed, reducing the data transfer costs

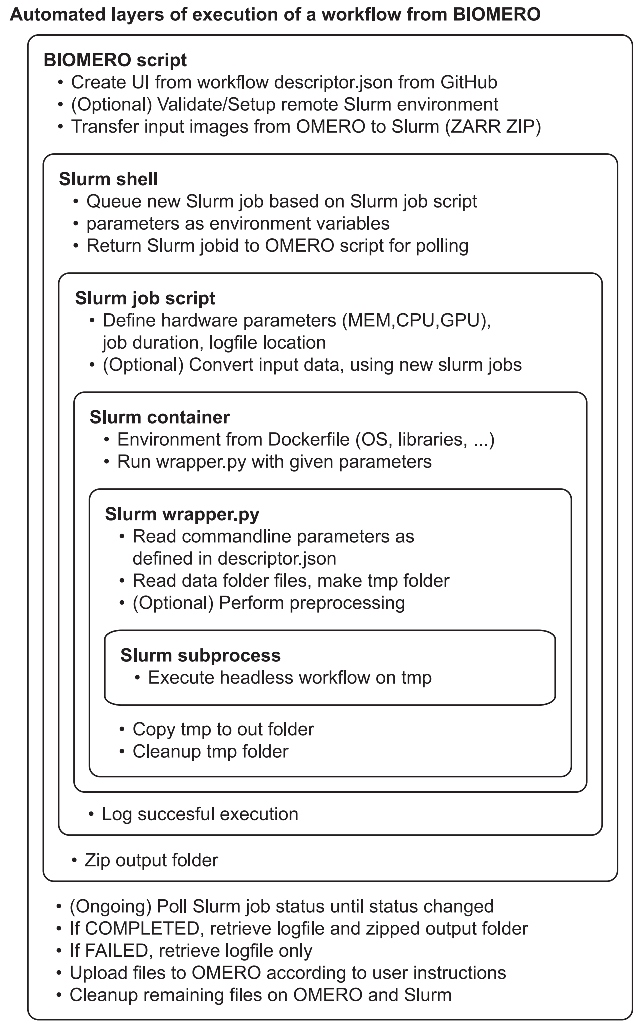

Figure 3. A workflow execution from BIOMERO will use a lot of automated layers of execution

It will start at the OMERO script "*SLURM Run Workflow*" that will allow a user to select a workflow with its parameters (defined in descriptor.json on GitHub). Once selected, the OMERO script will export the selected input data (dataset, plate, or just a set of images) as ZARR files and wrap them up in a ZIP. The ZIP will be transferred to a Slurm node. The script will then order the workflow (preinstalled on Slurm) to be run. This will trigger a command on the Slurm's Linux shell to queue a new job based on a (preinstalled) job script and environment variables. The Slurm job script will first define the hardware configuration required for this workflow (GPU, CPU, etc.) but also a predefined execution duration and the location of the log file. Next, it could trigger new Slurm jobs to convert all ZARR data to TIFF files and await their execution, or this conversion can be executed beforehand by the BIOMERO script. Once completed, it will start a Singularity container for the workflow (preinstalled on Slurm from Docker) and await its completion. The container will have an installed environment (OS, libraries, etc.) defined in its Dockerfile, required for the workflow to execute properly. When run, the container will call upon an entrypoint script called wrapper.py (also defined in its Dockerfile and on GitHub). This wrapper script, based on BIAFLOWS, will read and validate the required parameters (defined in descriptor.json on GitHub) from the command line. Furthermore, the wrapper will handle any preprocessing required (e.g., cut images into smaller sizes) and transfer data to a temporary folder before it will start a sub-process with the actual executable code (Fiji Macro, MATLAB executable, Python or R script, CellProfiler pipeline, etc.). After execution is finished, generated output files will be moved to the "out" folder, the temporary folder will be cleaned up, and Slurm will be informed of a successful execution. The OMERO script will poll the Slurm cluster for status updates during this execution and start extracting output and log data when it finds the job completed. Once the data are back on the OMERO server, it will upload them into the OMERO system based on user-defined preferences (e.g., as images in a new dataset or as a ZIP attachment for a project). Finally, all intermediate data will be cleaned up, and the OMERO.web UI will be informed of a successful script execution (with appropriate logging messages).

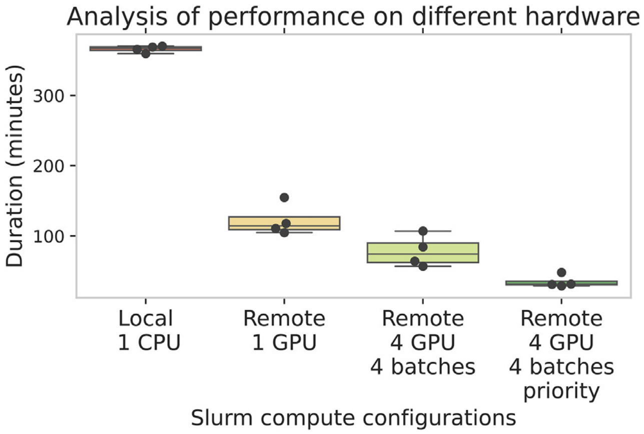

Figure 4. Comparison of computation times on Slurm with different compute configurations

We show the computation time (in minutes) for workflow CellPose (https://github.com/TorecLuik/W_NucleiSegmentation-Cellpose) on dataset S-BSST265[13] (duplicated 12 times for a total of 948 images) for different Slurm configurations: local 1 CPU, remote 1 GPU, and 2 variants with 4 batches each, remote 4 GPU 4 batches and remote 4 GPU 4 batches priority. The local 1 CPU is executed on our workstation with local Slurm containers (https://github.com/Cellular-Imaging-Amsterdam-UMC/NL-BIOMERO-Local-Slurm; no GPU support) and mimics running CellPose locally after downloading data from OMERO. Note that it is not just 1 CPU but 1 CPU node (with 4 CPU cores and 5 GB memory). The remote 1 GPU is executed on our remote HPC cluster with default BIOMERO settings for CellPose (1 job on 1 GPU node). The remote 4 GPU 4 batches are using the batch BIOMERO script to automatically run 4 jobs with 1/4th of the data each (manually chosen) in parallel on our remote HPC cluster, including practical delays like a batch waiting in the Slurm queue for other (people's) jobs. Finally, remote 4 GPU 4 batches priority is a theoretical extrapolation where we show the maximum time of any of the batches as the job's duration, a scenario that would be possible on an HPC cluster with 4 available GPUs. The batches were limited to 4 since our HPC account has a limitation of 4 GPUs in parallel, so in other HPC scenarios, the computation could speed up even more by making smaller batches. We show a steady decrease in workflow computation time by leveraging all facets of BIOMERO and Slurm together. The raw experimental data behind the boxplots are shown in Table S1.

significantly, so in the future, we might only have to transfer a link pointing to the original file (given that the Slurm cluster can access this endpoint too). This will reduce the strain on the framework and OMERO server.

Finally, we will develop a reactive UI that allows easier access to the workflows, replace the OMERO.scripts UI, and make workflows more findable by integrating with existing workflow registries such as WorkflowHub.eu[19] or the BioImage Model Zoo.[20] Within OMERO, a user should also be able to search for an existing workflow, or, in other words, workflows should be more findable and accessible.

EXPERIMENTAL PROCEDURES

Resource availability

Lead contact

Further information and requests for resources should be directed to and will be fulfilled by the lead contact, Przemek M. Krawczyk (p.krawczyk@amsterdamumc.nl).

Materials availability

This study did not generate new unique reagents.

Data and code availability

- This paper analyzes existing, publicly available data. The data are available in the BioImage Archive (http://www.ebi.ac.uk/bioimage-archive) under accession number S-BSST265.
- All original code for the BIOMERO Python library has been deposited at Zenodo under https://doi.org/10.5281/zenodo.8108214 and is publicly available as of the date of publication at GitHub (https://github.com/NL-BioImaging/biomero) and PyPI (https://pypi.org/project/biomero) under the permissive Apache 2.0 (https://www.apache.org/licenses/LICENSE-2.0) license.
- We also provide the example BIOMERO scripts (https://github.com/NL-BioImaging/biomero-scripts) repository, allowing execution of 2D image-to-image workflows directly from the OMERO web interface script interface as described in this paper. These are in a separate repository on GitHub with the more restrictive copyleft GPL-2.0 (https://www.gnu.org/licenses/old-licenses/gpl-2.0.html) license, as parts are copied from OME's GPL-2.0 licensed work, and can be installed directly on the OMERO.server to get started with BIOMERO; see the READMEs on GitHub for more details.

SUPPLEMENTAL INFORMATION

Supplemental information can be found online at https://doi.org/10.1016/j.patter.2024.101024.

ACKNOWLEDGMENTS

This publication is part of the project NL-BioImaging-AM (with project number 184.036.012) of the National Roadmap research program, which is (partly) financed by the Dutch Research Council (NWO). Special thanks to our collaborators within the NL-BioImaging project for their valuable contributions. We also appreciate the support and insights provided by the bioimaging community and OME team on image.sc, which greatly enhanced the quality and scope of this research. Icons used in images are (adapted) from Material Design Icons (https://pictogrammers.com/library/mdi/) and OpenClipart (https://openclipart.org).

AUTHOR CONTRIBUTIONS

Conceptualization, T.T.L., E.A.J.R., R.A.H., and P.M.K.; methodology, T.T.L., P.M.K., and R.A.H.; software, T.T.L.; validation, R.R.-B.; writing – original draft, T.T.L.; writing – review & editing, P.M.K., R.A.H., E.A.J.R., and R.R.-B.; funding acquisition, E.A.J.R.; supervision, P.M.K. and R.A.H.

DECLARATION OF INTERESTS

The authors declare no competing interests.

DECLARATION OF GENERATIVE AI AND AI-ASSISTED TECHNOLOGIES IN THE WRITING PROCESS

During the preparation of this work, the authors used ChatGPT in order to improve readability and language of the text and generate drafts for paragraphs. After using this tool/service, the authors reviewed and edited the content as needed and take full responsibility for the content of the publication.

Received: January 9, 2024
Revised: March 26, 2024
Accepted: June 18, 2024
Published: July 18, 2024

REFERENCES

1. Allan, C., Burel, J.-M., Moore, J., Blackburn, C., Linkert, M., Loynton, S., MacDonald, D., Moore, W.J., Neves, C., Patterson, A., et al. (2012). OMERO: flexible, model-driven data management for experimental biology. Nat. Methods *9*, 245–253. https://doi.org/10.1038/nmeth.1896.

2. Schindelin, J., Arganda-Carreras, I., Frise, E., Kaynig, V., Longair, M., Pietzsch, T., Preibisch, S., Rueden, C., Saalfeld, S., Schmid, B., et al. (2012). Fiji: an open-source platform for biological-image analysis. Nat. Methods *9*, 676–682. https://doi.org/10.1038/nmeth.2019.

3. Bankhead, P., Loughrey, M.B., Fernández, J.A., Dombrowski, Y., McArt, D.G., Dunne, P.D., McQuaid, S., Gray, R.T., Murray, L.J., Coleman, H.G., et al. (2017). QuPath: Open source software for digital pathology image analysis. Sci. Rep. *7*, 16878. https://doi.org/10.1038/s41598-017-17204-5.

4. Wilkinson, M.D., Dumontier, M., Aalbersberg, I.J., Appleton, G., Axton, M., Baak, A., Blomberg, N., Boiten, J.-W., da Silva Santos, L.B., Bourne, P.E., et al. (2016). The FAIR Guiding Principles for scientific data management and stewardship. Sci. Data *3*, 160018. https://doi.org/10.1038/sdata.2016.18.

5. Barker, M., Chue Hong, N.P., Katz, D.S., Lamprecht, A.-L., Martinez-Ortiz, C., Psomopoulos, F., Harrow, J., Castro, L.J., Gruenpeter, M., Martinez, P.A., and Honeyman, T. (2022). Introducing the FAIR Principles for research software. Sci. Data *9*, 622. https://doi.org/10.1038/s41597-022-01710-x.

6. de Visser, C., Johansson, L.F., Kulkarni, P., Mei, H., Neerincx, P., Joeri van der Velde, K., Horvatovich, P., van Gool, A.J., Swertz, M.A., Hoen, P.A.C.'., and Niehues, A. (2023). Ten quick tips for building FAIR workflows. PLoS Comput. Biol. *19*, e1011369. https://doi.org/10.1371/journal.pcbi.1011369.

7. Marée, R., Rollus, L., Stévens, B., Hoyoux, R., Louppe, G., Vandaele, R., Begon, J.-M., Kainz, P., Geurts, P., and Wehenkel, L. (2016). Collaborative analysis of multi-gigapixel imaging data using Cytomine. Bioinformatics *32*, 1395–1401. https://doi.org/10.1093/bioinformatics/btw013.

8. Rubens, U., Mormont, R., Paavolainen, L., Bäcker, V., Pavie, B., Scholz, L.A., Michiels, G., Maška, M., Ünay, D., Ball, G., et al. (2020). BIAFLOWS: A Collaborative Framework to Reproducibly Deploy and Benchmark Bioimage Analysis Workflows. Patterns *1*, 100040. https://doi.org/10.1016/j.patter.2020.100040.

9. Seiffarth, J., Scherr, T., Wollenhaupt, B., Neumann, O., Scharr, H., Kohlheyer, D., Mikut, R., and Nöh, K. (2024). ObiWan-Microbi: OMERO-based integrated workflow for annotating microbes in the cloud. SoftwareX *26*, 101638. https://doi.org/10.1016/j.softx.2024.101638.

10. Luik T.T., Rosas-Bertolini R., Reits E.A.J., Hoebe R.A., Krawczyk P.M. (2024). BIOMERO: BioImage Analysis in OMERO. Zenodo. https://doi.org/10.5281/zenodo.12191646.

11. Glatard, T., Kiar, G., Aumentado-Armstrong, T., Beck, N., Bellec, P., Bernard, R., Bonnet, A., Camarasu-Pop, S., Cervenansky, F., Das, S., et al. (2017). Boutiques: a flexible framework for automated application integration in computing platforms. Preprint at arXiv. https://doi.org/10.48550/arXiv.1711.09713.

12. Stringer, C., Wang, T., Michaelos, M., and Pachitariu, M. (2021). Cellpose: a generalist algorithm for cellular segmentation. Nat. Methods *18*, 100–106. https://doi.org/10.1038/s41592-020-01018-x.

13. Taschner-Mandl, S., Ambros, I.M., Ambros, P.F., Beiske, K., Hanbury, A., Doerr, W., Weiss, T., Berneder, M., Ambros, M., Bozsaky, E., et al. (2020). An annotated fluorescence image dataset for training nuclear segmentation methods. https://www.ebi.ac.uk/biostudies/bioimages/studies/S-BSST265.

14. Hartley, M., Kleywegt, G.J., Patwardhan, A., Sarkans, U., Swedlow, J.R., and Brazma, A. (2022). The BioImage Archive - Building a Home for Life-Sciences Microscopy Data. J. Mol. Biol. *434*, 167505. https://doi.org/10.1016/j.jmb.2022.167505.

15. Crusoe, M.R., Abeln, S., Iosup, A., Amstutz, P., Chilton, J., Tijanić, N., Ménager, H., Soiland-Reyes, S., Gavrilović, B., Goble, C., and Community, T.C. (2022). Methods included: standardizing computational reuse and portability with the Common Workflow Language. Commun. ACM *65*, 54–63. https://doi.org/10.1145/3486897.

16. Köster, J., and Rahmann, S. (2012). Snakemake—a scalable bioinformatics workflow engine. Bioinformatics *28*, 2520–2522. https://doi.org/10.1093/bioinformatics/bts480.

17. Di Tommaso, P., Chatzou, M., Floden, E.W., Barja, P.P., Palumbo, E., and Notredame, C. (2017). Nextflow enables reproducible computational workflows. Nat. Biotechnol. *35*, 316–319. https://doi.org/10.1038/nbt.3820.

18. Moore, J., Allan, C., Besson, S., Burel, J.-M., Diel, E., Gault, D., Kozlowski, K., Lindner, D., Linkert, M., Manz, T., et al. (2021). OME-NGFF: a next-generation file format for expanding bioimaging data-access strategies. Nat. Methods *18*, 1496–1498. https://doi.org/10.1038/s41592-021-01326-w.

19. Goble, C., Soiland-Reyes, S., Bacall, F., Owen, S., Williams, A., Eguinoa, I., Droesbeke, B., Leo, S., Pireddu, L., Rodríguez-Navas, L., et al. (2021). Implementing FAIR Digital Objects in the EOSC-Life Workflow Collaboratory (Zenodo). https://doi.org/10.5281/zenodo.4605654.

20. Ouyang, W., Beuttenmueller, F., Gómez-de-Mariscal, E., Pape, C., Burke, T., Garcia-López-de-Haro, C., Russell, C., Moya-Sans, L., de-la-Torre-Gutiérrez, C., Schmidt, D., et al. (2022). BioImage Model Zoo: A Community-Driven Resource for Accessible Deep Learning in BioImage Analysis. Preprint at bioRxiv. https://doi.org/10.1101/2022.06.07.495102.

Patterns, Volume *5*

Supplemental information

BIOMERO: A scalable and extensible image analysis framework

Torec T. Luik, Rodrigo Rosas-Bertolini, Eric A.J. Reits, Ron A. Hoebe, and Przemek M. Krawczyk

Figure S1. Slurm folder structure, related to BIOMERO Python library

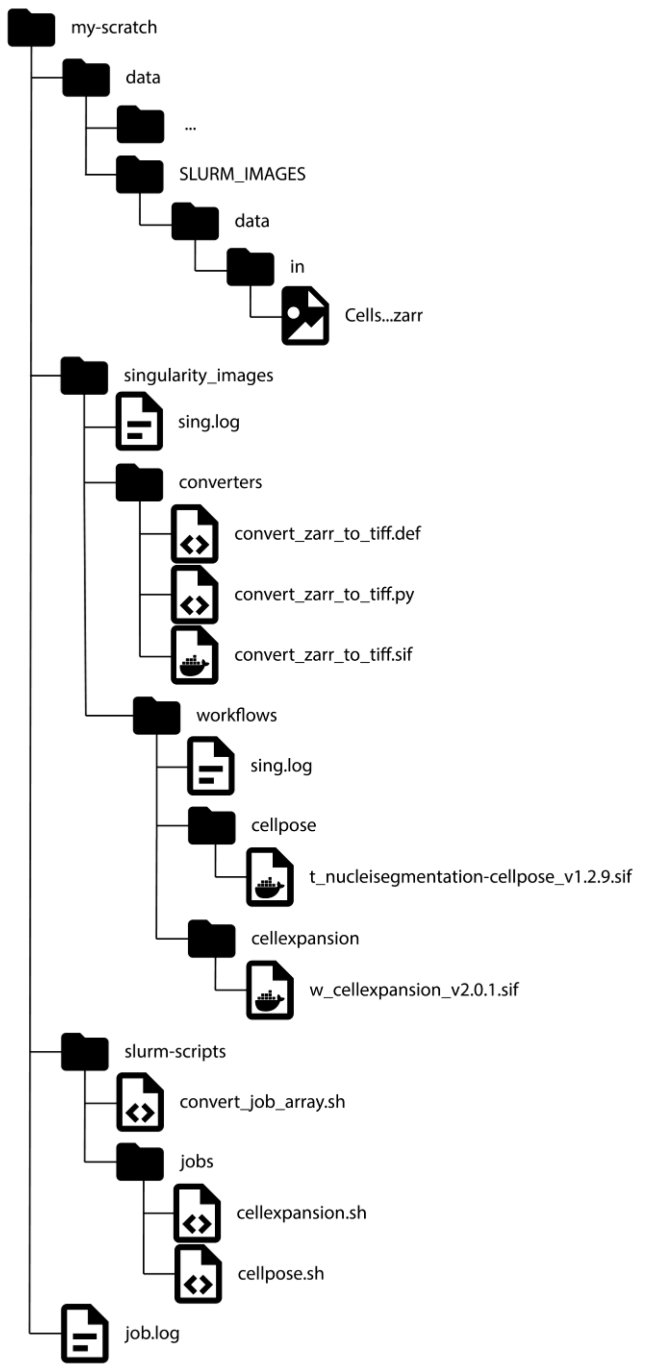

Figure S1 – Folder structure on Slurm nodes managed and created by BIOMERO. By design, all Slurm nodes share a storage in a folder ('my-scratch'), where logs of the running jobs are written. BIOMERO populates this storage with 3 folders: "singularity_images", "slurm-scripts" and "data". First

and foremost, “singularity_images” contains all container images for all workflows and converters, downloaded in the Singularity Image Format (SIF). “slurm-scripts” contains a Slurm job script for each workflow in the jobs subfolder. This folder could be pulled from Git instead of generated. Finally, “data” will contain the actual datasets transferred from OMERO (as zip files). Data will be unpacked into a folder structure required for BIAFLOWS workflows, with subfolders in(put), out(put) and g(round)t(ruth); only ‘in’ is shown. All these elements are automatically set up and can be refreshed by BIOMERO at any time when creating a connection.

Note S1. OME-NGFF, related to BIOMERO scripts and web interface.

For workflows to be FAIR, it is important to have 1 type of data (image) input. The Cytomine-0.1 apps from BIAFLOWS all expect TIFF files as input (and output). However OME is working on the OME-NGFF[1] (ZARR) to be a future data standard for bioimaging, especially designed for image data analysis. To support both directions, we are exporting all selected images from OMERO to the ZARR format, using the OMERO-CLI-ZARR (https://github.com/ome/omero-cli-zarr) app. On the Slurm cluster we run a converter from ZARR to TIFF format, using Slurm Array (https://slurm.schedmd.com/job_array.html) capabilities to parallelize and queue the extra computation on the high-performance hardware. This way we support current Cytomine-0.1 apps with TIFF but also open the door to future apps using ZARR. This is an extendable design where future workflows could use other converters, or (preferably) work directly on the ZARR data.

Figure S2. Dataset in OMERO, related to Example Use Case

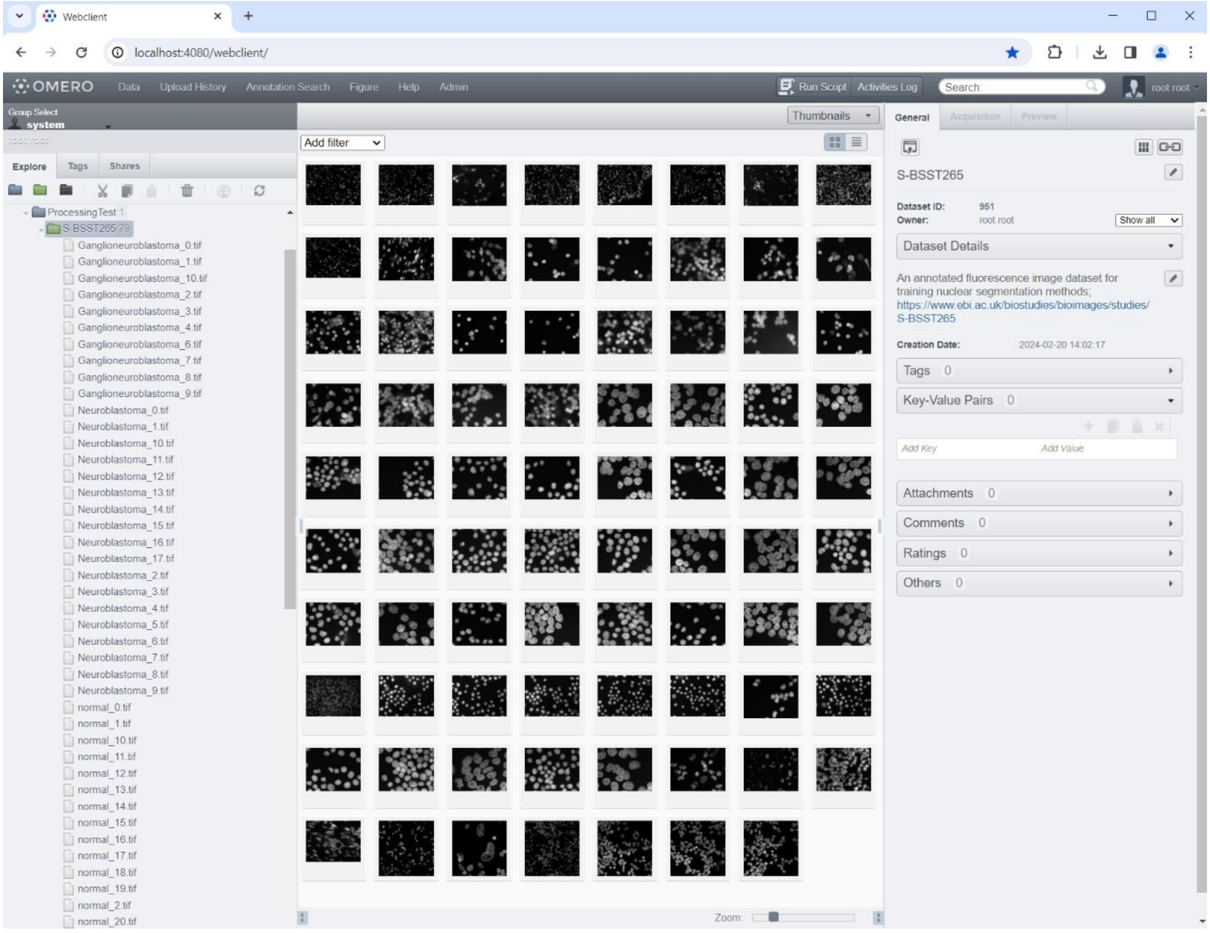

Figure S2 – Screenshot of the OMERO user interface, with (the 79 raw images of) dataset S-BSST265 imported into a dataset. Ready to start analyzing with BIOMERO.

Figure S3. Select BIOMERO script in OMERO, related to Example Use Case

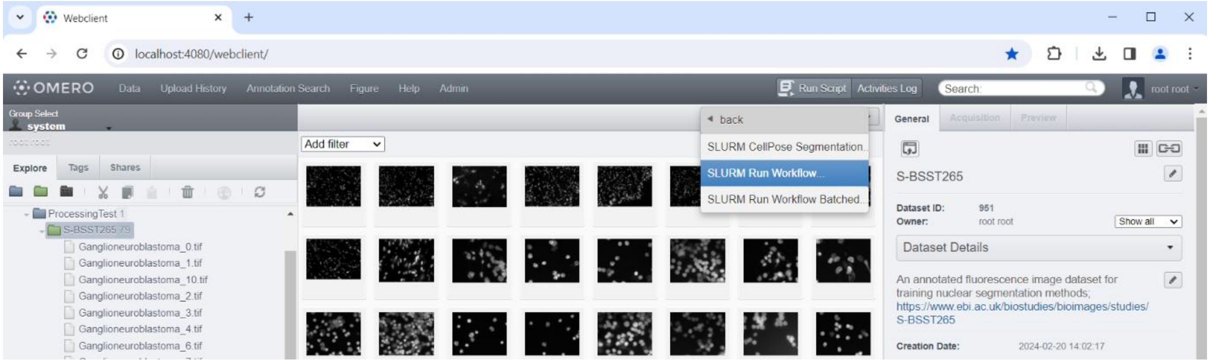

Figure S3 – Screenshot of the OMERO user interface, after clicking the Run Script button (note, this button is a cogwheel icon in default OMERO) and navigating to BIOMERO > workflows > SLURM Run Workflow. Note that you can preselect the dataset (shown in the left panel) to have it automatically selected as input data for the (BI)OMERO script.

Figure S4. BIOMERO script UI in OMERO, related to Example Use Case

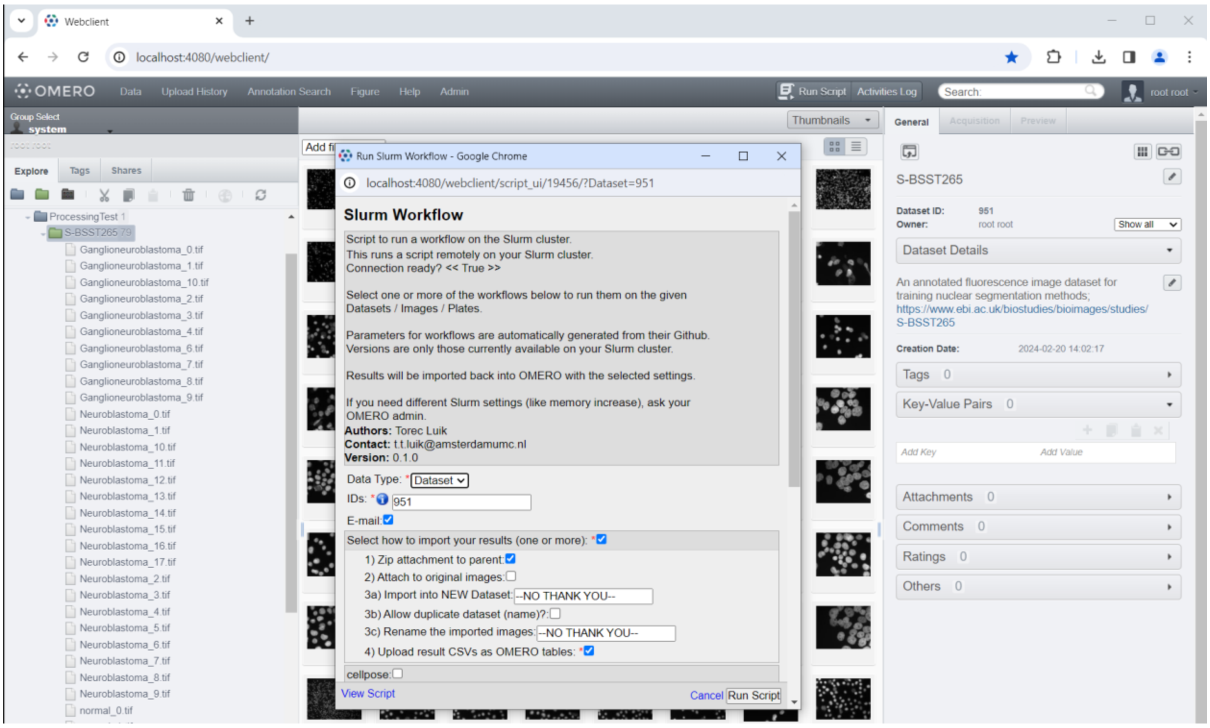

Figure S4 – Screenshot of the OMERO user interface, after clicking the SLURM Run Workflow script option. The preselected dataset is added as input ID and the pop-up user interface shows options for how the user wants to import results back into OMERO. Further down the UI are the options for the configured BIOMERO workflows, here starting with ‘cellpose’.

Figure S5. Larger BIOMERO script UI in OMERO, related to Example Use Case

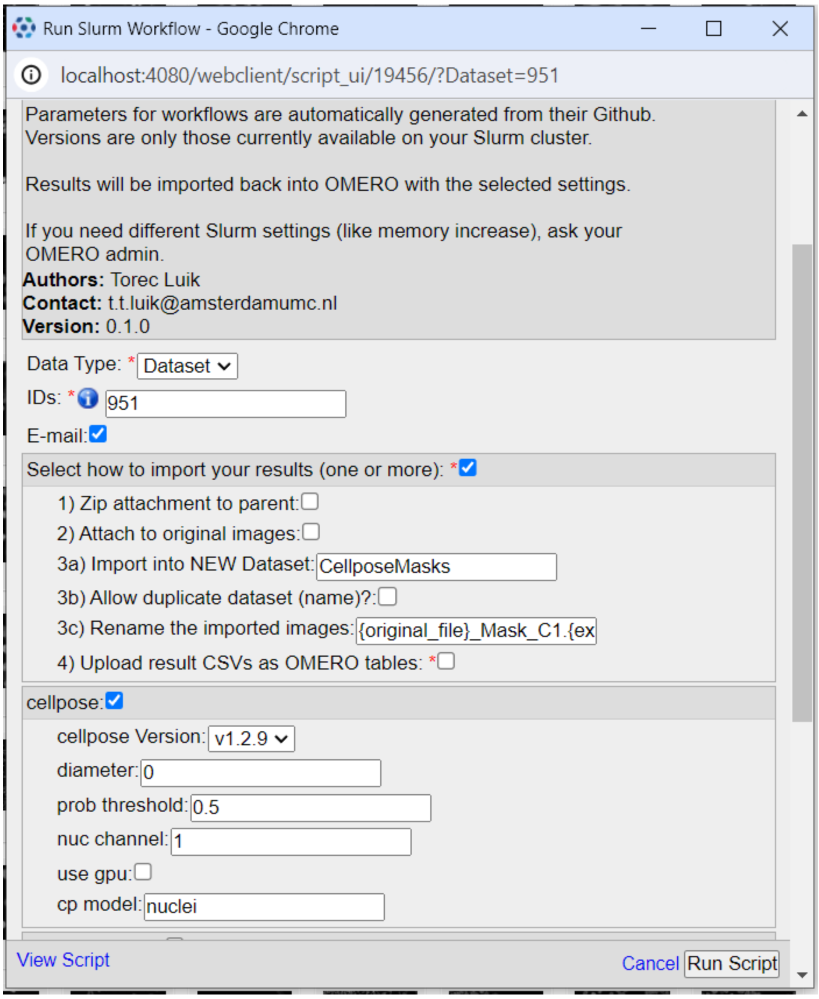

Figure S5 – Screenshot of the OMERO user interface, after clicking the SLURM Run Workflow script option, now zoomed in more. The preselected dataset is added as input ID and the pop-up user interface shows options for how the user wants to import results back into OMERO. We have selected to import the result (mask images from CellPose) into an OMERO dataset named 'CellposeMasks' and to rename these results to reference the image they originated from. Further down the UI are the options for the configured BIOMERO workflows, here we selected 'cellpose' and it showing all the parameters that are predefined in Cellpose's descriptor.json on its GitHub repository (generated on-the-fly). The version shown (v1.2.9) is a selection of container versions available on the connected Slurm cluster (queried when generating this user interface). We have selected to run CellPose on

channel 1 with the default ‘nuclei’ model, default threshold and default diameter (CellPose will guess the nucleus diameter per image), but not using GPU.

Figure S6. Processing BIOMERO script in OMERO, related to Example Use Case

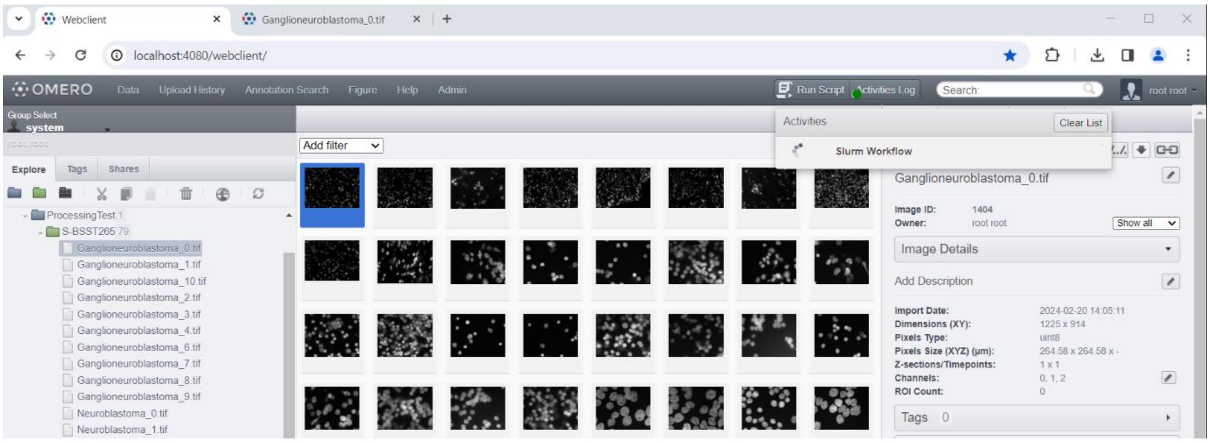

Figure *S6* – Screenshot of the OMERO user interface, after running the SLURM Run Workflow script from Figure S5. In the background the script will perform all required operations, but the user will only see the rotating icon in the activity window until the workflow is done (completed, or erroneous).

Figure S7. Results of BIOMERO script in OMERO, related to Example Use Case

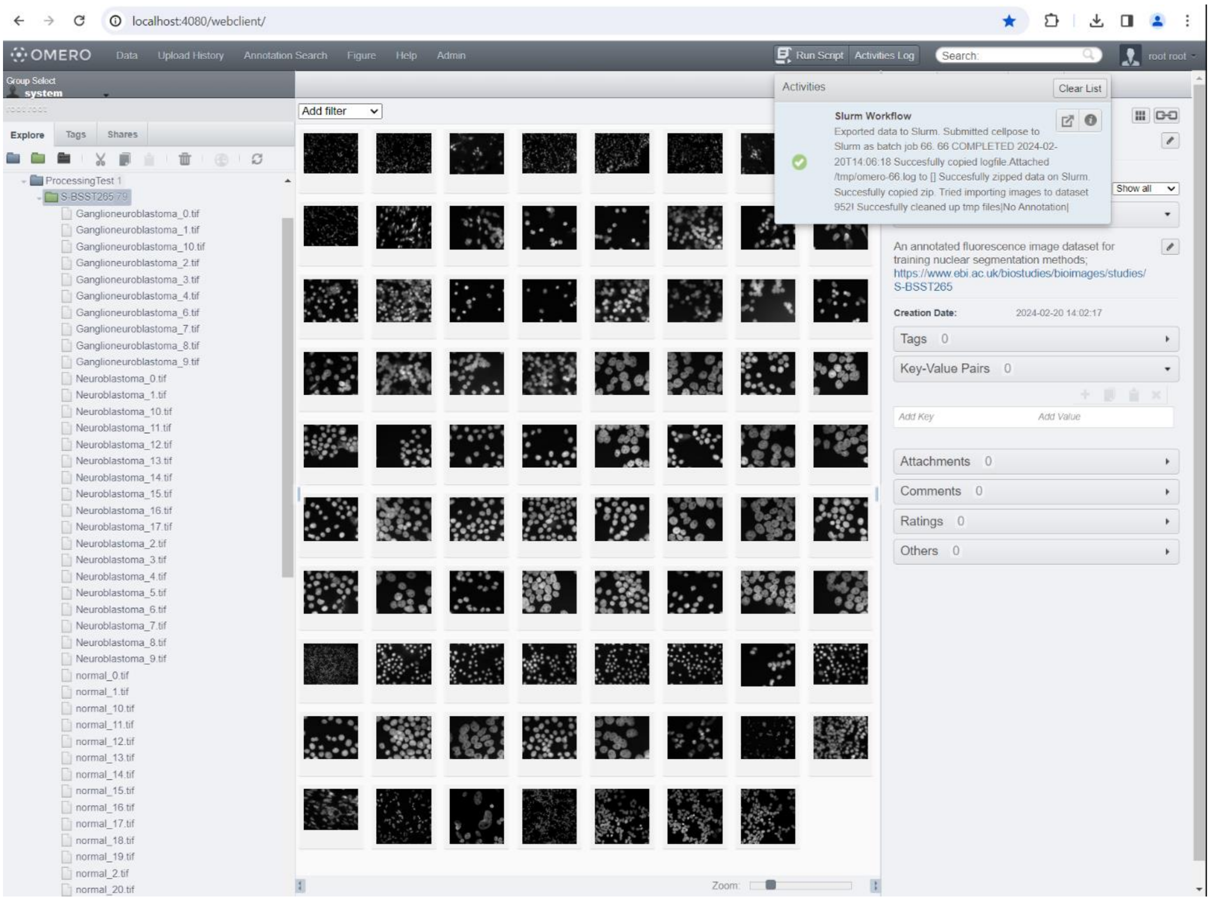

Figure S7 – Screenshot of the OMERO user interface, after running the SLURM Run Workflow script from Figure S5 and processing in Figure S6. A summary log is shown in the activity window, with a more detailed log attached from the BIOMERO script at the 'I' button. This is standard OMERO script behavior.

Figure S8. Resulting dataset of BIOMERO script in OMERO, related to Example Use Case

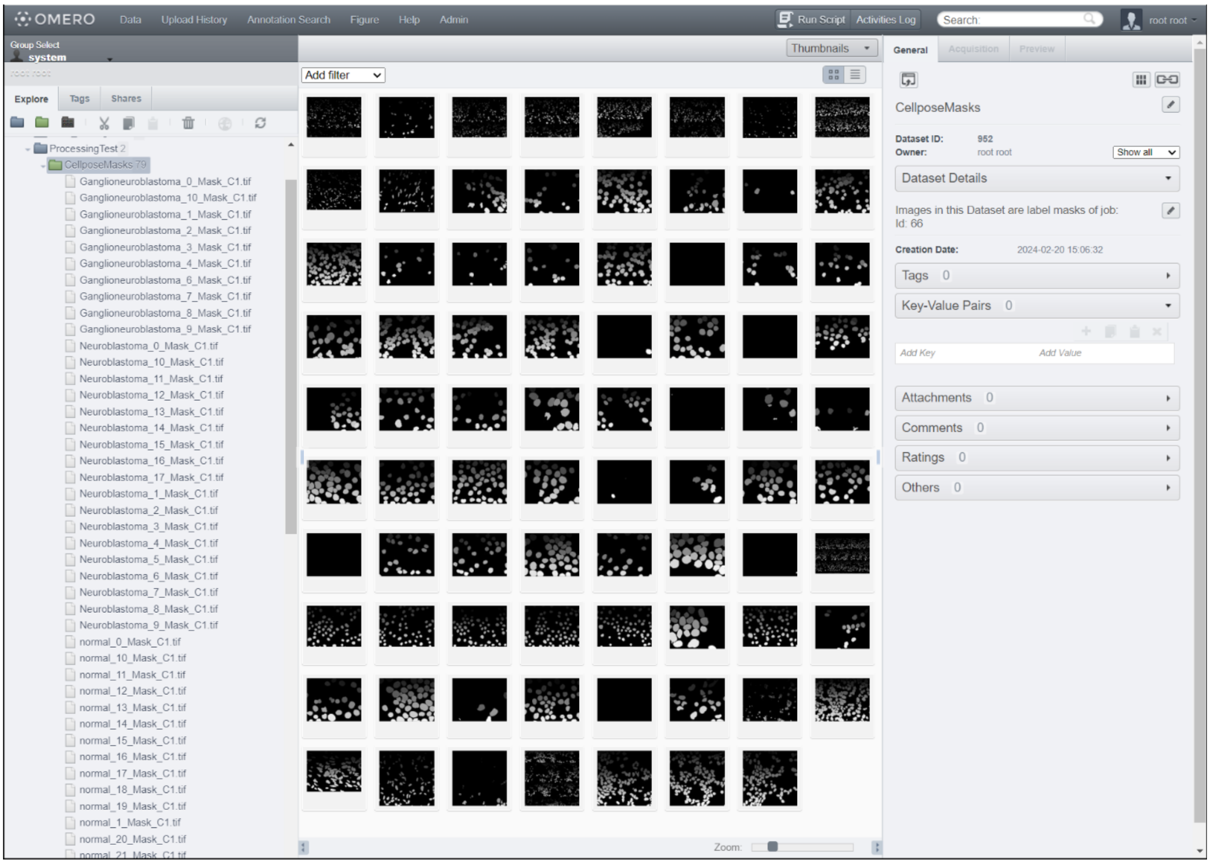

Figure S8 – Screenshot of the OMERO user interface, after the SLURM Run Workflow script from Figure S5 is completed successfully. A new dataset has been added (shown in the left panel), with all the mask images created by the CellPose workflow uploaded and renamed to match their origin image.

Table S1. Computation duration per job, related to Figure 4

Compute Configuration	Job id	Subjob	start	end	duration	Priority duration	Duration (min)	Priority duration (min)
Local 1 CPU	4308		14:00:58	20:06:28	06:05:30		365.5	
	5257		11:35:03	17:34:32	05:59:29		359.48	
	6206		10:10:23	16:19:14	06:08:51		368.85	
	7155		10:17:20	16:27:23	06:10:03		370.05	
Remote 1 GPU	265755		15:37:18	18:11:58	02:34:40		154.67	
	272945		13:54:49	15:45:29	01:50:40		110.67	
	275113		09:39:25	11:24:16	01:44:51		104.85	
	280745		11:16:16	13:14:04	01:57:48		117.8	
Remote 4 GPU 4 batches	264077	1	14:50:51	15:26:10	00:35:19		35.32	
		2	14:55:41	15:21:23	00:25:42		25.7	
		3	14:59:51	15:36:21	00:36:30		36.5	
		4	15:06:56	15:55:00	00:48:04		48.07	
		Total	14:50:51	15:55:00	01:04:09	00:48:04	64.15	48.07
	266608	1	09:40:10	10:08:05	00:27:55		27.92	
		2	09:43:43	10:06:07	00:22:24		22.4	
		3	09:47:27	10:17:09	00:29:42		29.7	
		4	10:06:09	10:37:10	00:31:01		31.02	
		Total	09:40:10	10:37:10	00:57:00	00:31:01	57.0	31.02
	271249	1	11:56:48	12:25:25	00:28:37		28.62	
		2	12:13:18	12:37:02	00:23:44		23.73	
		3	12:32:20	13:01:35	00:29:15		29.25	
		4	12:49:48	13:21:17	00:31:29		31.48	
		Total	11:56:48	13:21:17	01:24:29	00:31:29	84.48	31.48
	273440	1	07:42:50	08:09:29	00:26:39		26.65	
		2	08:09:51	08:31:46	00:21:55		21.92	
		3	08:31:51	09:00:04	00:28:13		28.22	
		4	09:00:51	09:29:50	00:28:59		28.98	
		Total	07:42:50	09:29:50	01:47:00	00:28:59	107.0	28.98

Table S1 – Computation duration per job on different Slurm configurations. The start and end are queried from the Slurm accounting ('sacct') information and the durations and minutes are calculated from these times. The ‘Remote 4 GPU 4 batches’ jobs have subjobs that are indicated with ids 1,2,3,4 and are aggregated in 2 different ways in the ‘Total’ row: the duration between the start of the first job and end of the last job; and the duration of the longest of its subjobs (called the Priority duration). The Boxplot in Figure 4 shows the distribution of these job durations in minutes, with batches only using the Total row and splitting out duration and priority duration into 2 boxes.

Supplemental References

1. Moore, J., Allan, C., Besson, S., Burel, J.-M., Diel, E., Gault, D., Kozlowski, K., Lindner, D., Linkert, M., Manz, T., et al. (2021). OME-NGFF: a next-generation file format for expanding bioimaging data-access strategies. Nat Methods *18*, 1496–1498. 10.1038/s41592-021-01326-w.
